# Henry W. Williams, A. M., M. D.

**Published:** 1895-09

**Authors:** 


### Obituary.

### HENRY W. WILLIAMS, A. M., M. D.

THIS eminent ophthalmologist and author, whose fame was not confined to this country, but extended throughout Europe, has been a conspicuous professional figure for many years. He was born in Boston, on December 11, 1821, and Boston continued to be his home during his whole life. He was educated in the schools of his city and prepared to enter Harvard college at the Latin School. His health, however, was so poor that he did not enter college. He took up mercantile pursuits, instead, but these became distasteful to him and he soon abandoned them and began the study of medicine. He attended lectures at the Harvard Medical School and graduated in 1849. He then went to Europe for three years, following the various hospitals and the services of distinguished practitioners in Berlin, Heidelberg, Zurich and Paris. Much of his time was spent in the latter city, and it was here that he became especially interested in diseases of the eye. On his return to Boston he began the practice of medicine in all its various departments. Ere long, however, he drifted into his chosen specialty and was soon prominent as an ophthalmic surgeon. In 1864, the Boston City Hospital was organised and he was made its ophthalmologist, a position which he held till 1891, when, by reason of ill-health, he resigned and was made consultant. In 1867, he was foremost in establishing the Massachusetts Medical Benevolent Society, which was designed for the relief of professional men and their families. He was afterward its president for many years. He was an active Fellow of the Massachusetts Medical Society from 1849 and was its president in 1880 to 1882. He was also an early member (1864) of the American Ophthalmological Society and was its president in 1869, 1870, 1871 and 1872. Harvard, in 1868, conferred upon him the honorary degree of A. M. Three years later he was made an honorary member of the Phi Beta Kappa society. In 1868, also, he won the Boylston prize for an essay entitled Recent Advances in Ophthalmic Science. The year was made still more eventful to him by a call which he received from Harvard Medical School to the chair of ophthalmology. This position he occupied for twenty-two years, after which, on account of ill-health, he retired. But on his retirement he endowed the chair generously.

He was the author of numerous writings, among which are: Our Eyes and How to Use Them; Recent Advances in Ophthalmic Science; and Diagnosis and Treatment of Diseases of the Eye. His last public appearance of note was, last spring, before the American Academy of Arts and Sciences, of which he was a member, when he read an obituary of the late von Helmholtz.

Dr. Williams was a man of large stature and strong character. He was an excellent teacher, being clear, forcible and persuasive in his speaking. As an operator on the eye, he was ambidextrous, and is said to have had few equals. He achieved great success in his profession, and was an example worthy of imitation in his untiring patience, punctuality and conscientious devotion to duty.

Dr. Williams died at his home, 15 Arlington street, Boston, June 13, 1895, after a very short illness. He leaves a widow, one daughter and six sons, three of whom are in the medical profession. One of the latter, Dr. Charles H. Williams, is well known as an ophthalmic practitioner and assisted in the revision of the enlarged edition of his father's work on diseases of the eye, one of the best manuals on the subject which has ever emanated from the American profession.

The remains of Dr. Williams were tenderly borne to their last resting-place by his sons. Thus closes the last chapter in the life of another one who has made invaluable contributions to the world and left an indelible impress upon this generation by his example and his works.

